# Impact of Authoritative and Laissez-Faire Leadership on Thriving at Work: The Moderating Role of Conscientiousness

**DOI:** 10.3390/ejihpe11030048

**Published:** 2021-07-05

**Authors:** Zulfiqar Ahmed Iqbal, Ghulam Abid, Muhammad Arshad, Fouzia Ashfaq, Muhammad Ahsan Athar, Qandeel Hassan

**Affiliations:** 1 School of Business Administration, National College of Business Administration and Economics, Lahore 54660, Pakistan; z.aiqbal@yahoo.com (Z.A.I.); fouziams@hotmail.com (F.A.); ahsan.athar@list.edu.pk (M.A.A.); syeda.qandeel28@gmail.com (Q.H.); 2 Department of Business Studies, Kinnaird College for Women, Lahore 54660, Pakistan; ghulam.abid@kinnaird.edu.pk

**Keywords:** authoritative, laissez-faire, thriving, conscientiousness

## Abstract

This study empirically investigates the less discussed catalytic effect of personality in the relationship of leadership style and employee thriving at work. The growth and sustainability of the organization is linked with the association of leadership style and employee thriving at the worplace. The objectives of this study are to explore the impact of authoritative and laissez-faire leadership styles and the moderating role of the personality trait of conscientiousness on thriving in the workplace. A sample of 312 participants was taken from a leading school system with its branches in Lahore and Islamabad, Pakistan. The participants either worked as managers, teachers in headquarters, or school campuses, respectively. The regression results of the study show that authoritative leadership and conscientiousness have a significantly positive impact on thriving at work. Furthermore, conscientiousness moderates the relationship between laissez-faire style of leadership and thriving at work relationship.. The findings of this study have theoretical implications for authoritative and laissez-faire leadership, employee conscientiousness, and managerial applications for the practitioners.

## 1. Introduction

Educational institutes are idiocentric hubs that preserve and promote cultural values to the next generation [1]. Numerous previous studies have discussed the effectiveness of leadership style in different perspectives. Scholars have noticed that the role of leadership style has been extensively explored and discussed from the business perspective; however, its vital importance in the background of educational centers responsible for providing country’s future leadership was less investigated during the last few decades [2]. They are convinced that the dream of sustainable development remains ineffective without active participation and thriving of employees at the workplace, which is promoted by the practicing organization leadership style [3]. This study empirically explores the role leadership style in promoting employee thriving at the workplace in the background of educational system in the developing countries, particularly in the context of Pakistan. 

As the school system is a complex organization, practicing interactive functions, multifarious human resource, and objectives [4], scholars are agreed that recent years have brought challenging intricacies to the school leaderships [5], and leadership style has an explicit connection to the modern challenges of educational institutes. In this context, leadership style motivates the followers to achieve the organizational objectives [6]. Practicing leadership style is an integral factor to address the modern intricacies of the educational system. The cordial relationship between leadership and employees on the principals and business process ensures the successful achievement of an organizational goal [5]. Therefore, an effective leadership enhances and maintains the competitive advantage of an organization [7]. However, the effectiveness of leadership is based on employee willingness and cooperation to execute the orders [8]. In connection with leadership, it is significant to develop understanding of employee thriving at work, which refers to a positive psychosomatic state characterized by a shared sense of vivacity and learning [9]. The collaborative impact of effective leadership [10] and thriving individuals [11] plays an important role in the growth of an organization. Being forward-thinking and maintaining a competitive advantage are the keys to sustain the growth of an organization. Scholars and researchers link organizations’ success with individuals thriving [11]. Thriving is promoted by an individual’s characteristics [12], such as conscientiousness. Organizations need to look after their human capital and their well-being; as a result, they will flourish in the workplace. Although the role of thriving has been demonstrated by previous studies [11], during the few decades, the catalytic factor of employees’ conscientiousness, the relationship of leadership style, and employee thriving at work remained less explored. Therefore, this study is designed to empirically investigate the impact of authoritative and laissez-faire leadership styles on employee thriving. This study also discusses the moderating factor of employee’s conscientiousness in the relationship of leadership styles and employee thriving.

This research demonstrates that leadership style has a direct association with thriving at work. It also shows that employee personality factor plays a significant role in promoting thriving. The current study is significant for both academia and practitioners. The researchers are interested in investigating the antecedents of thriving. This research highlights three important aspects: firstly, that a leader can adopt a style to enhance thriving at work, and secondly, that relevant personality traits can be identified and practiced to increase thriving. Lastly, it provides insight into how a combination of effective leadership style and personality traits can significantly impact workplace thriving and employees’ health and well-being.

Organizations need to grow and thrive in very competitive business environments. In this context, employee motivation and thriving at the workplace depends a lot on the manager’s style of leading [13]. Their leadership style and the typical personality traits exhibited by them influence the motivation of their subordinates [11]. An organization with a culture of values and a mission provides grounds for motivation and thriving in its employees [13]. The modern workforce is very diverse, which enhances the importance of leaders’ relationships with their workers. This leads toward the requirement that a leadership style matches the needs of the situation and the characteristics of subordinates. This also highlights the need for sound personality traits possessed by a leader, which would enhance thriving at work.

Thriving has been defined as an individual’s capacity to prosper, grow, flourish, and develop vigorously in the workplace. It is a psychological phenomenon [14] and amounts to experiencing learning and vitality at work. Both learning and vitality enhance each other [15]. Scholars have observed that people who thrive at work are relaxed and secure and feel cherished [14]. They are also convinced that thriving employees at work are generally more energetic [16]. Thriving employees exhibit a desirable behavior in the workplace, such as innovation and organizational commitment, as well as less burnout [11,17,18].

There has been a continuous effort on the part of researchers to identify a style of leadership that better impacts a follower’s behavior and attitudes [13]. Various leadership styles have been dominant in organizational behavior, the most prominent being transformational and charismatic leadership [19]. The new dominant theories of leadership styles are authentic, servant, and ethical leadership. For the purpose of this research, we focus on authoritative and laissez-faire styles of leadership. 

Previous research has explored that the authoritative leadership style is considered best when an organization is drifting. They argue that authoritative leaders have a vision and enjoy the ability to articulate direction to people toward it [20]. Those who work under an authoritative leader understand the need and importance of what they do and why. The expected rewards and laid-down procedures/standards are also clear to them. In times of uncertainty, authoritative leadership is sought by people [11]. Authoritative leaders are result oriented and they make all decisions, accomplish tasks, and use penalties and punishment rather than rewards to achieve the desired results in the stipulated amount of time and resources [21].

Laissez-faire is another leadership style, which is derived from the French term that means “to let it do” [21]. In most management and leadership styles, employee participation is of paramount importance. The laissez-faire style is considered to be at the extreme end of the democratic-style spectrum [21]. Laissez-faire leaders delegate decision-making powers to followers. This process creates good learning opportunities for followers [22]. The style becomes more effective when employees are highly skilled and motivated [23], which is when it helps employees thrive at work.

Personality traits that influence leadership style are important factors that ultimately affect thriving at work. Conscientiousness explains the desire for accomplishment and its pursuit [24]. This trait is achievement-oriented and is equated with a sense of responsibility. Since the major theme of conscientiousness is achievement-oriented [25], it would be strongly linked and related to thriving in the workplace.

This is carried out in the background of the educational system of Pakistan. Researchers are agreed that leaders are not born, they are shaped by the educational and training institutes [26]. From this perspective, schools are the training centers where the seeds of constructive or destructive personalities are germinated [24]. They are also convinced that that good leadership is developed through an unending process of self-analysis, training and educating, and by accumulating a variety of experiences [24]. This study adds to the academic literature on the role of leadership styles, in connection with the employee thriving at the workplace. It draws the attention of managers and policy makers in the school system to explore the dimension of individual personality and understand the psychological factors that stimulate employee thriving at work. 

## 2. Literature Review

### 2.1. Theoretical Perspective

This study is based on the big five personality factor model that provides the basis for the assessment of personality characteristics in terms of their scores on five personality domains: (i) extraversion, (ii) emotional stability, (iii) agreeableness, (iv) conscientiousness, and (v) openness to experience [24]. These five factors of personality model have been tested as determinants [27] and performance indicators of leadership styles [28]. All the personality traits of the five factor personality model are integral factors for effective leadership styles [29]; however, the personality trait of ‘conscientiousness’ of leadership, which is associated with being thorough, organized, responsible and goal-oriented, is a prerequisite for organizational leadership for the promotion of employee thriving at the workplace [30]. Authoritative leadership refers to the command and confidence over followers, and it essentially holds the trait of conscientiousness for achieving the desired goal [31]. In contrast, the laissez-faire style of leadership empowers followers by involving them in decision making and instills in them the feeling of being integral components of the business process, which motivates them to direct their energies toward achieving the organizational objectives [32]. This discussion presumes that authoritative and laissez-faire leadership styles at educational institutes promote employee thriving at work.

### 2.2. Thriving at Work 

Thriving is a focus of researchers of organizational behavior [18,33]. Initially, thriving was considered a reaction to a challenge [34] that relates to growth. Thriving is an “individual’s experience of growth, development and progression with an upward trajectory, not merely serving or maintaining the status quo” [35]. It has been observed that employee thriving at work acts as a buffer against the negative psychological outcomes [16]. Learning is a very important dimension of thriving at work [36]. Vigor is another important component of thriving [37]. The work context shapes thriving, which is considered a psychological state but a temporary condition [14,16,37]. Thriving people feel a sense of progress [14]. Confidence is a trait of those who thrive at work, which also promotes self-regulation in employees. The thriving employee is capable of assessing their own development [38]. Many outcomes have been associated with thriving, such as physical and psychological well-being [16]. Increase in performance is also attributed to thriving [16]. The increased performance of employees at work promotes their sense of trust and connection with the organization [14].

Thriving indicates an increase in skills, knowledge, and confidence [34]. Thriving employees predict desirable outcomes such as innovation, job satisfaction, and organizational commitment [17,39]. Performance and thriving are positively related [16,17]. Performance is the appraisal and reward of a described job [40]. Resources such as better interpersonal relationships, meaningfulness, and knowledge at work are produced by thriving [14]. Thriving is considered to be important in challenging work situations [16]. Thriving workers recognize problems and deal with novel situations [39]. Thriving is a desirable state [14]. Organizations try to reduce turnover intention [41], and thriving plays a significant role in reducing this [42]. Thriving improves interpersonal relationships at work [18]. An employee’s engagement with the organization is enhanced by thriving [43]. The organization is benefitted through thriving individuals.

Individuals who thrive have been associated with sound psychological functions [16]. Thriving is enhanced by core self-evaluation [12]. Perceived support by the organization makes employees thrive [33,42]. Those individuals thrive more who possess a proactive personality [44]. Conscientiousness is positively related to thriving [45]. Empowered leaders motivate their followers by sharing power with them [46]. Thriving must be separated from resilience. Resilience means taking a position against difficult odds [47,48]. Thriving focuses on positive psychological work [14]. Thriving is different from flourishing, too. Flourishing means a mental state in which an individual is elevated by a sense of competence, positive relationship, and purpose of living [49], which is a relatively broad state that equips an individual to achieve a purpose of life. In contrast, thriving is associated with a subjective experience [14] and equates with a psychological state, being an intra-individual phenomenon. It is a combination of cognitive and affective dimensions of psychological experiences. Thriving has two important components, learning and vitality [14,16]. Individual learning with low vitality is not thriving. Conversely, vitality without learning is also not thriving. Thriving individuals are characterized by energy and greater psychological functioning [18]. It is also linked to physical health. Thriving is promoted by an organization sharing information about its overall strategies, feedback, and decision making.

Psychological capital also impacts workplace thriving [16]. It is viewed as a self-regulatory psychological state, whereas personality traits including core evaluation enhance employee thriving at work [38]. Employees thrive when they feel competent to do a task [33,42]. Individuals with a proactive personality thrive more [44]. Researchers have witnessed that extraversion and conscientiousness are positively related to thriving [45]. Psychologically safe environments provide employees with a sense of relatedness [50] and thriving. Psychological safety was linked to thriving [51] and an enriched work-family promotes thriving at work [52].

Thriving stimulates innovative work behavior [39,42]. Self-confidence is enhanced by learning, which facilitates innovative skills [39]. A sense of learning and vitality enhance commitment with the organization [14]. Those who learn continuously enjoy physical and mental health [53]. Thriving sustains an organization’s human resources. Thriving individuals are high performers and more committed [14]. Thriving promotes innovative behavior [39]. Employees interact with each other to perform their tasks. Their dedication and hard work contribute toward achieving the organization’s goals and result in gaining a competitive advantage. During their professional life, an individual may face work/family imbalance [42], hostile environments, and work stress. These factors, if unchecked, may lead to a dejected, non-thriving workforce [54]. Employees tend to thrive if they perceive they are supported by their organization. This enhances a positive relationship among coworkers [55,56]. A lot of work has been done on outcomes of positive relationships among coworkers [18].

Thriving stimulates innovation in the workplace [42]. Thriving enhances self-confidence and innovation [39]. Leaders inspire, motivate, and develop followers’ behavior and attitudes for thriving at work. Organizations need to grow and thrive in a very competitive business environment and this depends on having a dedicated and highly motivated workforce [11]. Employee motivation to develop and thrive depends a lot on their leaders, the leadership style used, and the personality traits exhibited by them [13]. Such employees see themselves as valuable assets of the organization. The leadership team provides the path and the methods of achieving goals to its followers.

Previous studies have observed that in the recent past, due to rapid industrialization and globalization, it has become increasingly important for the organization to maintain employee thriving at work to meet the competitive advantage [57]. Pressure and learning, exerted by the leadership, are the two dimensions of employee thriving. Employee thriving is linked with the prevailing leadership style in the organization [58]. The impact of leadership style on the employees is discussed in the following sections. 

### 2.3. Authoritative Leadership and Thriving at Work

There have been continuous efforts in the past on the part of researchers to identify the style of leadership that would impact the subordinate’s behavior and attitudes positively [14]. Previous studies have explored that various styles of leadership have been dominant in an organization, the most prominent being transformational and charismatic styles [19]. An effective style of leadership is the main source of competitive advantage [59]. An organization grows and performs well if the leadership is effective [10]. Leaders are perceived and accepted by followers as leaders [59]. “Leadership is a management function which is directed toward people and social interaction, as well as on the process of influencing people so that they will achieve the goals of the organization” [60].

Authoritative leadership is defined as commanding and self-confident. Authoritative leaders earn respect and are obeyed. An authoritative leader motivates employees by enforcing discipline and rigid rules and procedures. It is used where strict compliance is needed and no error could be tolerated. Effective authoritative leadership style influences employee thriving at work. The research on leadership is very rich [61]. This finding was further confirmed by scholars [62,63]. Later research found that uncertainty calls for the authoritative style of leadership. Authoritative leaders serve to reduce uncertainty [64]. 

There are many leadership styles being used by leaders. The authoritative leadership style is most prevalent in a Chinese setting. Authoritative leadership is defined as “one element of paternalistic leadership [31]. This type of leadership is assertive, has control over followers, and commands obedience” [11]. An authoritarian leader enjoys asymmetric power and control over others and exercises dominance by strict rules and uses the threat for deterring insubordination [65]. They are a strict disciplinarian and exert authority in decision making. Subordinates of authoritative leaders may experience negative emotions toward the leader [61]. Authoritative leaders often overrule followers’ suggestions, which may make them resentful toward them [11]. Employees may feel a violation of the psychological contract. “Psychological contract is an individual’s belief of an agreement‘s terms between the individual and the organization” [66]. Mutual obligation is the basic concept behind it. Authoritative leaders may intimidate subordinates into obedience [67], which may cause anger and fear.

Employees thriving at work predict desirable outcomes related to innovative behavior and commitment to the organization. Employee thriving in previous studies has been discussed with servant leadership [68], transformational leadership [69], authentic leadership [70], and leader–member exchange [71]. Authoritative leaders are high on demand and expect compliance; they focus on procedures. Their standards and expectations are high but they are also responsive and warm. They are good listeners too and are supportive. An authoritative leader gives a lot and expects a lot. They exercise authority appropriately and in a timely manner. They emphasize professional learning. Authoritative leadership positively affects thriving at work. Therefore, this study proposes the following hypothesis:

**Hypothesis** **1** **(H1):** *Authoritative leadership is positively related to thriving at work.*

### 2.4. Laissez-Faire and Thriving at Work

Laissez-faire is another leadership style that is passive compared to authoritative leadership [21], which has been noted as more active and assertive in the previous discussion. Scholars have explored laissez-faire and perceived it as an ineffective style [32]. They are convinced that laissez-faire leaders let their followers have liberty in decision making, and this type of latitude promotes followers’ belief of efficacy [72]. Previous studies have explored that leaders should perceive and demonstrate according to the situation so that followers may be perceived as empowered as compared to laissez-faire leadership [73]. It has also been observed that followers have their own expectations from a leader; therefore, when a leader’s behavior matches the follower’s expectations, they are more elevated and their performance serves as more effective [74]. Contrary to the above, ineffective participation of leaders influences the follower’s perception of laissez-faire leadership. Once the perception is of ineffectiveness is developed among the subordinates, the leader may not be able to motivate them to perform as per market demand [21].

Empowering leadership is characterized by involving employees in decision making [75]. Empowering leadership of this kind is perceived as positive [76] and an active leadership style [77]. A laissez-faire leader is seen as ineffective [78], and they avoid interaction and problems; this creates a perception of ineffectiveness. Laissez-faire leaders are considered as failing to handle responsibilities [22]. Laissez-faire is termed as non-influencing [79]. It is also equated with non-leadership. It is also defined as an ineffective and inactive leadership style. The laissez-faire style abdicates legal powers [21,32]. Followers perceive leaders according to their own needs and expectations. This style is known to avoid responsibilities. They exercise little control over their followers. The laissez-faire style can be very effective if followers are highly skilled and motivated [23]. These leaders let followers make their own decisions.

Leadership is one of the most observed and least understood phenomena on earth. Leadership creates vision and enriches subordinates [80]. The leadership style largely depends on the culture of the organization and the situation. The laissez-faire style gives maximum freedom to employees. The leaders do not exercise direct supervision. Laissez-faire leadership may not always be attributed to avoiding or being insensitive to followers’ needs. Subordinates are expected to be monitored but, at times, they would like to be left alone. Laissez-faire leaders allow space to followers for innovation. Laissez-faire leadership needs to be approached in a balanced way. “Laissez-faire leadership is considered to be ineffective or zero leadership”. Such views prevail because laissez-faire has not been researched in-depth [79]. Some empirical studies suggest positive outcomes of laissez-faire leadership, such as innovation. “Leadership is how a leader is perceived by followers” [81]. Laissez-faire leadership should be approached in a balanced way and considered in a more neutral manner.

Laissez-faire leadership should be considered as non-involvement. Non-involvement does not mean being inactive [79]. It could be perceived as respect for an individual’s personal competence [82]. It could also be viewed as avoidance of imposing the self by the leader. It removes bureaucratic restraints; non-involvement and empowering leadership could be seen as the same [83]. The non-involvement of laissez-faire leadership and empowering leadership are similar [77]. A non-involved laissez-faire leader could be viewed as a substitute for leadership. According to the substitute theory, certain characteristics of an employee or situation affect a leader’s ability to affect employees [84]. The theory of substitution endorses situational factors for the effectiveness of laissez-faire leadership.

Laissez-faire leaders allow subordinates to make decisions [85]. The leaders normally avoid imposing on a group’s activities. A previous study has explored that when managers do not take timely action, it can affect subordinates’ efficiency negatively [86]. Laissez-faire is seen as an absence of leadership. Empirical studies on the laissez-faire style of leadership have concentrated more on its relationship with employee satisfaction with their job and the desired outcome; it was found to be negatively correlated with job satisfaction [78].

The laissez-faire style of leadership delegates decision-making power to subordinates. It gives freedom to subordinates to decide their own activities related to tasks. The leaders provide the necessary support. This process provides a good learning opportunity to followers [22]. This style proves very effective when employees are highly skilled and motivated. However, this style is unsuitable for subordinates who lack skill and knowledge. The involvement of a leader may have unintended negative effects on employees’ needs. The unnecessary involvement of a leader may cause negative effects. Reinforcement theory supports the administration of rewards or punishment, but this could lead to an undermining of reasoning and conduct [87] and can increase dependency in followers. It can also result in a negative influence on creativity [88]. Previous study shows that an introvert may often be a more effective leader in dynamic situations (reinforcement threatens the sense of self-competition) [89]. Laissez-faire leadership creates feelings of self-regulation among followers [90]. They allow the followers to have self-control and increase their self-efficacy [22]. The followers have a sense of empowerment [12], self-regulation, and leadership. Laissez-faire leadership provides autonomy to its followers [91]. This autonomy leads to psychological empowerment and self-leadership [92]. With this argument, laissez-faire leadership may facilitate an innovative environment [93], which helps workers thrive. Laissez-faire leadership style in the light of the above argument is perceived as ineffective and an avoidance of taking responsibility, whereas employee thriving is characterized as dealing with pressure and maintaining the pace of learning. Therefore, the laissez-faire style of leadership is expected to negatively influence employee thriving at work. This discussion is hypothesized as follows:

**Hypothesis** **2** **(H2):** *Laissez-faire leadership style is negatively related to thriving at work.*

### 2.5. Conscientiousness and Thriving at Work

Conscientiousness, also called the ‘will to achieve’ [94], is equated with dependability [95]. It is also termed as thoroughness, being organized, and responsible. Being achievement-oriented and perseverance are attributed to conscientiousness. Conscientiousness refers to goal-directed behavior, self-discipline, and persistence [96]. Conscientiousness is more achievement-oriented and not ethical [25].

Personality psychology helps with understanding the whole person [97]. Individuals differ from each other in traits and behavior. The components of personality need to be explained. The Cybernetic Big Five Theory (CB5T) provides a framework of personality. Human behavior is an outcome of the person and their environment. Traits have relatively less effect on complex social behavior [98]. Conscientious individuals are found to perform better and are achievement-oriented [99]. They are ambitious [100]. Conscientious individuals are self-focused and self-governing. They are found to be reliable and dependable [99]. Such persons set goals for themselves and are committed to achieving them [101].

The personality traits of a leader affect their effectiveness considerably. There is a general lack of agreement on trait terms [102]. Trait concepts are needed in personal selection, job counseling and in many other situations. The psychometric approach is agreed by most researchers for using or defining traits in personality. For the purpose of this paper, the trait of conscientiousness is selected, as it has a profound influence on thriving at work. Conscientiousness explains the desire for the pursuit [100]. Since a major theme of conscientiousness is achievement-oriented behavior [25], it is expected to be strongly linked to thriving at work. Personality is one of the most important topics and predictive of many life dimensions [103]. Several models have been used to describe personality. The big five model is one of the most well-regarded personality models [99]. The big five model describes individual differences. The factors addressed by the big five model are neuroticism, extraversion, openness to experience, agreeableness, and conscientiousness. This model has been widely used for classifying individual differences [104].

The big five model explains five personality traits that enrich a leader’s personality and their effectiveness. The personality traits are defined as “People’s stylistic habitual pattern of cognition, affect and behavior” [105]. Personality traits are associated with individuals. The big five model has been specified as the abridged big five dimensional circumplex model [106]. Five traits are described in the big five model: “extraversion, agreeableness, conscientiousness, emotional stability, and intellectual autonomy” [25,100]. The ABC model was developed on the basis of the factor analysis of a large number of traits: descriptive effectiveness. Self-legislation is an important factor of the human personality that is included from the perspective of a model of personality traits, the five-factor model as discussed earlier [99]. Conscientiousness means controlling one’s behavior in pursuit of one’s goals. Self-regulation is associated with goal-attainment. Self-regulation can also be termed as self-control. People with high self-control are termed as conscientious. Conscientious individuals have a high level of organization and order; it also improves time management [107]. Conscientious individuals set high standards for themselves and also exhibit a high level of commitment. They have control over their habits or impulses and display self-disciplined. 

Since the conscientiousness trait of personality controls one’s behavior, it serves as an antecedent factor to influence the pressure and learning process in terms of employee thriving at work [17]. Therefore, it is believed that the conscientious personality traits of an employee positively influence thriving at work. In addition, authoritative leadership motivates employees by enforcing discipline, which continuously sustains pressure on employees to keep on learning by executing the given task within defined timelines [24]. The presence of the conscientiousness personality trait further stimulates the desire of pursuing the goals that ease the authoritative leadership to achieve the organizational goals. Therefore, it is supposed that the moderating effect of conscientiousness as an employee personality trait promotes the relationship between authoritative leadership style and employee thriving at work. Extending the discussion, the laissez-faire leadership style disowns the power, and an employee is at liberty in working and decision making [30]; therefore, the employee’s pace of learning will be reduced, and a conscientiousness employee feels no pressure to achieve the timelines, which further lessens the employee commitment [27]. Thus, all these factors influence thriving at work.

**Hypothesis** **3** **(H3):** *Conscientiousness is positively related to thriving at work.*

**Hypothesis** **4** **(H4):** *Conscientiousness moderates the relationship between authoritative leadership and thriving such that the relationship is positive for conscientious individuals.*

**Hypothesis** **5** **(H5):** *Conscientiousness moderates the relationship between laissez-faire and thriving such that the relationship is negative for conscientious individuals.*

**Figure 1:**
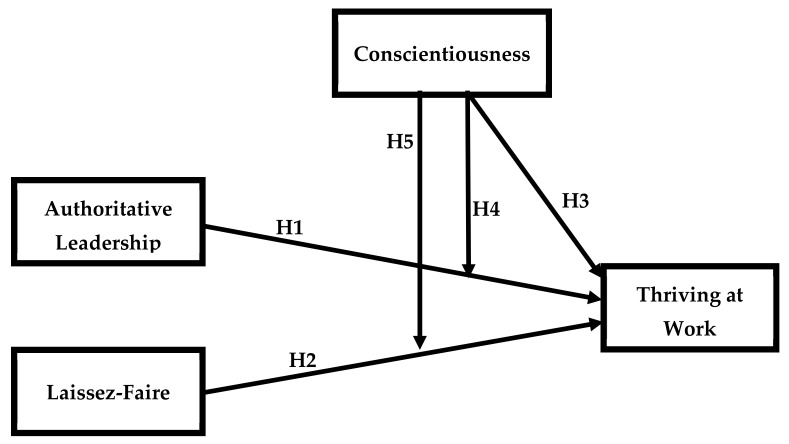
Theoretical Model.

This study is anchored in big five model of personality traits [99], which explains the comparative impact of authoritative and laissez-faire leadership styles on employee thriving at work. This model explains the five traits of a leaders’ personality including extraversion, agreeableness, conscientiousness, emotional stability, and intellectual autonomy” [25,100]. This research is an effort to investigate the moderating impact of the “conscientiousness trait” in the relationships that authoritative and laissez-faire leadership styles have with employee thriving. Since the big five model of personality traits inscribes all the possible behaviors of a leader that affect the employee–leadership relationship, therefore, its application in the underlying study would possibly produce valuable findings that would add to the academic literature and also assist practitioners in the Pakistani education system and other business enterprises. The conscientiousness personality trait of leadership has the ability to control the behavior of employees; therefore, this was expected to moderate the relationship of leadership style and employee thriving at work. In the view of the above discussion and the previous literature review, the following framework is proposed (see Figure 1).

## 3. Methodology

### 3.1. Procedure and Sample

We tested our hypotheses in a major school system with its branches in the major cities of Pakistan. We selected its branches in Lahore and Islamabad for our study. The reason for choosing these cities was that the data were collected from the head office of that school system, and they were located in the specified cites. Lahore and Islamabad are considered to be the hub of major business activities in this region, particularly in the educational system [108]. Therefore, data were collected from these major educational cities. Prior to data collection, we coordinated a visit to the head office in Lahore. We met the concerned senior management and explained the purpose of our research. We also assured them that we would keep the confidentiality of their respondents. They agreed to extend their full cooperation to facilitate our research. After having sought their permission and cooperation, we initiated the process of data collection. Data were collected using the purposive sampling technique. Data were collected from 200 respondents from the head offices situated in Lahore and Islamabad of major schools system by using a questionnaire, which is the most commonly used tool to collect data from respondents for quantitative studies [109]. The questionnaires were delivered and collected physically from Lahore and through the mail from Islamabad. The survey questionnaires distributed in Lahore were collected on the same day. We received the same from Islamabad within a fortnight; a total of 312 survey forms were duly completed and received from both cities. The reasons for selecting this particular sample were threefold. Firstly, the population selected was education-related, which is relevant to the variables under study. Secondly, the willing cooperation of the management was a welcome facilitator that made it possible to get timely responses. Thirdly, the educated participants could easily understand the questions and give appropriate responses.

### 3.2. Measures 

In this study, we measured the variables of authoritative leadership, laissez-faire, and conscientiousness in context with employee thriving at work. The effect of conscientiousness as a moderator on authoritative leadership and thriving and on laissez-faire leadership and thriving was also examined. The participants were selected from a leading school system. The sample was collected from the head offices in Lahore and Islamabad. Both managers and teachers were targeted for the research. For each survey item, a five-point Likert-scale was used. The scales ranged from 1 (strongly disagree) to 5 (strongly agree). For thriving, a scale developed by scholars [16] was used. The calculated internal consistency of the scale was 0.80. The scale was used for measuring conscientiousness, which is one of the personality traits in the big five model, which was developed by scholars [109]. The conscientiousness variable is measured using the personality traits model because conscientiousness is a personality construct that is a core determinant of health, positive aging, and human capital, and almost all the studies use the big five model to assess conscientiousness. The calculated internal consistency of the scale was 0.90. The leadership style test (authoritative and laissez-faire) was developed in the form of the multi-factor leadership questionnaire (MLQ). 

### 3.3. Control Variables

We used five demographic variables for understanding their influence on the relationship between authoritative leadership, laissez-faire leadership, and conscientiousness on thriving at work. Control variables were used to test the accuracy value of an independent variable on the dependent variable. It is a variable that does not change its value throughout the study, which also allows the researcher to better understand the relationship among other variable that are tested. We focused more on age, education, and experience, as these have profound effects on the outcomes. With age come maturity and understanding and an increased sense of responsibility. Education enhances awareness, knowledge, and promotes rationalization. Experience has always been considered a founding stone for commitment, organizational identity, and productivity. All these variables contribute to thriving at work.

## 4. Results

Data Analysis

The data of the current research study were analyzed using SPSS-24. Mean and standard deviations of the demographic and study variables were analyzed using descriptive statistics. A reliability test was performed to ensure that the study variables are reliable to perform further analysis. Correlation analysis of the demographics and study variables was also performed in order to check the association among demographics and study variables and study variables with each other. Regression analysis was performed to check the direct relationship among study variables. For moderation analysis, Process by Hayes was used, and Model 1 was applied to assccess the role of the moderator among on the relationships among the independent and dependent variables of the study.

Table 1 provides the mean, SD of the results, and the correlation analysis. It also shows that the majority of the respondents were an average age of about 33 years, were married, had a graduate education level, and had job experience of more than four years. The mean responsiveness of the respondents about authoritative leadership, laissez-faire leadership, conscientiousness, and thriving at work was above 3.20. Reliability analysis of the study variables is above the standard of 0.70. Cronbach’s alpha reliability scores were authoritative (0.83), laissez-faire (0.79), conscientiousness (0.94), and thriving at work (0.83). Authoritative leadership is significantly related to thriving at work (r = 0.19, *p* < 0.01). Laissez-faire is significantly and negatively associated with thriving at work (r= -0.14, *p* < 0.05) and negatively related with conscientiousness. Conscientiousness is also significantly positively associated with thriving at work (r = 0.13, *p* < 0.05).

Table 2 shows the regression analysis of the direct relationships of our study variables. In Model 1, the relationship of the demographic variables with thriving is checked, which shows that all of them have non-significant relationships with thriving and the r-square is very low, which means that the demographic variables only contribute a 1% change in thriving. In the same way, in Model 2, when we apply the authoritative leadership style, it is significant (β =0.14 **, *p* < 0.01) and contributes 2% variation to thriving as it was proposed in H1 hypothesis. Therefore, results affirms our supposition that authoritative leadership has significant positive association with employee thriving at work. Furthermore, in Model 3, we test the relationship of the laissez-faire leadership style with the demographic variable that shows a significant contribution (β= −0.13 *, *p* < 0.05), and causes 2% variation in thriving, that agains affirm our assumption of hypothesis (H2). The results of H1 and H2 indicate low variance in the employee thriving by both leadership styles, which suggests that each hypothesis should be retested by conducting another study to validate the findings. In Model 4, conscientiousness is significant (β= 0.09 *, *p* < 0.05) and creates a 6% variation in thriving, which shows that conscientiousness has a significant positive association with thriving at work (H3). In Model 5, hierarchical regression is calculated, and it shows that authoritative, laissez-faire, and conscientiousness are significantly associated with thriving and collectively contriute 39% variation in thriving.

Table 3 shows the regression analysis of the proposed moderations through using Process by Hayes. The results show a direct and significant relationship of conscientiousness (β = 0.84 **, *p* < 0.01) and laissez-faire (β = 0.72 **, *p* < 0.01) with thriving at work. The results also indicate that the interactional term of laissez-faire × conscientiousness is also significant (β= -0.23 **, *p* < 0.01). This shows that conscientiousness moderates the relationship between laissez-faire and thriving at work; therefore, H5 of our study is being supported. In contrast, the results show that the interactional effect of authoritative leadership × conscientiousness is not significant (β= 0.07, *p* > 0.05). This shows that conscientiousness does not moderate the relationship of authoritative leadership and thriving at work; therefore, H4 of the study is not supported, as it was assumed in the literature.

**Figure 2:**
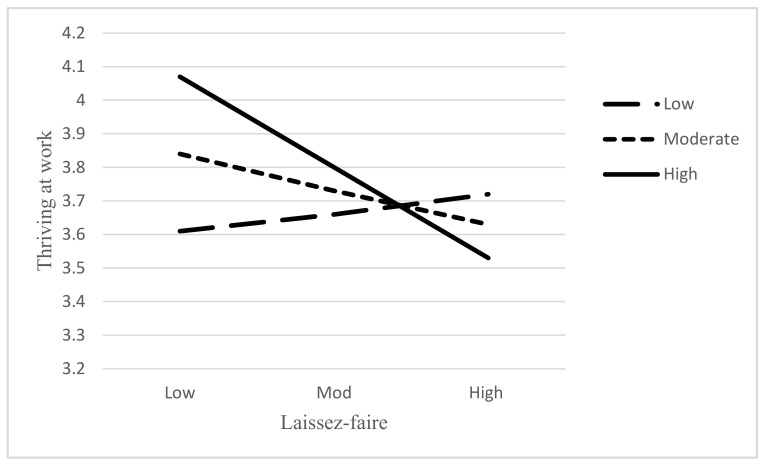
Moderation of conscientiousness on relationship between laissez-faire and thriving.

Figure 2 represents the graphical explanation of moderation. This shows that the relationship of laissez-faire and thriving at work is being influenced by the moderation of conscientiousness. It indicates that at a high level of conscientiousness, the relationship of laissez-faire and thriving at work has a stronger negative effect as compared to moderate and low levels of conscientiousness.

## 5. Discussion

Our study adds to the literature on the effects of authoritarian leadership and laissez-faire leadership styles on thriving at work, posing a new avenue of thought; i.e., the moderating effect of conscientiousness. The relationship between authoritarian leadership (H1) and laissez-faire (H2) leadership styles on employees’ thriving at work is revealed to positively influence their thriving. In contrast, the moderating role of conscientiousness on the relationship between authoritarian leadership and thriving is revealed to have a positive effect, which is interestingly opposite to our purposed hypothesis (H4), whereas the moderating effect of employee conscientiousness further strengthens the negative effect of laissez-faire style of leadership on thriving at work, as proposed by our study (H5). The results of the study are thought provoking. Therefore, the organizational management needs to understand the multidimensional roles of leaderships because they interact with diversified human resources at the workplace. The style of leaderships should reflect the objective of the organization, and developing sound understanding of their subordinates would help them achieve their organizational goals. Similarly, the employees’ conscientiousness trait would enable them to develop an advanced understanding of their prevailing leadership [110], which is again necessary to execute organizational objectives. It also signifies that the organization should understand the importance of the employee personality trait “conscientiousness” at the time of recruitment. The results are particularly more important since the educational system promotes conscientiousness among the students and the people engaged in promoting training activities. It draws the attention of institutional management to understand the role of leadership style in the educational system. The conscientiousness of employees rejects the passive leadership style that contributes insignificant impact while constituting policy reforms, and similarly, they also demonstrate indifferent behavior when their leadership suppressed their feeling. The leaders provide the necessary support that positively influences thriving. This non-involvement empowers individuals [83] and leads to positive outcomes. The results are also supported by the substitute theory [84]. The theory endorses that situational factors moderate the effectiveness of laissez-faire leadership. However, when we introduced conscientiousness as a moderator, we found that conscientiousness negatively moderates the influence of laissez-faire leadership on employee thriving. Moreover, the findings of this study suggest that authoritative leadership positively facilitates employee thriving when conscientiousness is introduced as a moderator. The study results reveal that conscientiousness is an achievement-oriented desire [25,100]. Conscientious individuals have a high level of organization and order and also improve time management [96,106]. Conscientious individuals set high standards for themselves; hence, conscientiousness as a moderator positively affects the authoritative leadership and employee thriving relationship.

### 5.1. Implications for Theory and Research

Thriving at work is an essential part of an employee’s work life that is affected by many factors. The style of leadership plays an important role in thriving at work. Authoritarian leadership positively influences thriving due to inherent assertiveness. Most workers need goading and pushing to thrive. The laissez-faire style is more acceptable to employees who prefer liberty in action and decision making and thrive on trust and a sense of responsibility. The personality factor plays a significant role in enhancing thriving at work. Conscientious workers do not want/need any authority to perform or thrive; they do it simply out of implicit motivation. As a moderator, conscientiousness shows a significantly negative effect on the relationship between both authoritative and laissez-faire leadership and thriving. 

Our results demonstrate that thriving is enhanced by using the right style of leadership, according to the motivation level and attitude of workers. Conscientious employees do not need any type of leadership to thrive at work. The right combination of leadership style and personality traits will be effective in enhancing thriving at work. The findings of this research encourage educational scientists to explore the role of leadership in the context of the big five model of personality traits other than conscientiousness in the context of mediation and moderation, since human behavior is complex and has an enormous impact in business as well as service industries including education, hospitality, banking, and consultancy services at different levels.

### 5.2. Implications for Practice

Organizations often work on the premise of the positive-relationship approach only. This may not always work. At times, significantly negative relations may produce positive outcomes. In our study, the negative moderating effects of conscientiousness are an important indicator of thriving. Organizations may have limited control over workplace behavior. Our findings suggest that the authoritarian or laissez-faire approach may negatively affect thriving when moderated by conscientiousness. Instead of suppressing workplace behavior, organizations should employ practices to enhance the implicit motivation to thrive. The findings of this study suggest to the practitioners of the educational system to revisit the organizational policies in terms of hiring people in the supervisory role. The employees engaged in the educational system implicitly or/and explicitly influence the personalities of the little angels in our training centers who will design the country’s fate in the long run.

## 6. Limitations and Future Research

Our research is based on two types of leadership and one personality trait. Only one moderating variable is used. However, this study does not explore how the different types of leadership styles deal with a variety of human resources at an organization. More leadership styles and personality traits could be tested. The cultural impacts were also not addressed in this study. The sample size was small, and the number of participants could have been increased. The research was limited to samples from two cities only. The research reliance was placed on a cross-sectional study only due to constraints of time. Only one moderator was tested. It is felt that instead of investing in the development of new leadership theories, new behavior should be focused on enhancing thriving at work. The low variance (7% and 2%) in the results of H1 and H2 suggests that another study should be conducted to validate the impact of authoritative and laissez-faire leadership styles on employee thriving at work. Furthermore, an ideal combination of leadership and the right personality traits should be sought to increase thriving at work. As per this study, authoritarian and laissez-faire leadership styles are polar opposites of each other, but further studies can check whether these are polar opposites or not by testing in different regions and sectors. Cultural factors are little discussed in this study, and these should be included and further explored in future work for a better understanding of the phenomenon.

We suggest the relationship between other leadership styles (authentic, ethical, servant) and personality traits (openness to experience, agreeableness, and extraversion) should be explored while explaining how different types of leadership styles would be beneficial for the practitioners to deal with diversified human resources. This will help both employees and organizations maintain a productive and thriving workforce.

## 7. Conclusions

The focus of researchers lately has been on new styles of leadership in the context of organizations, whereas the importance and relevance of the original leadership styles remain unquestioned. Similarly, personality traits have their own significance in the effectiveness of a leadership style. This study highlights the importance and effectiveness of authoritative and laissez-faire styles of leadership in organizations and on thriving at work. Conscientiousness, as a personality trait, affects individuals’ thriving at work. We extended this study to a major school system where the results show a significantly positive relationship between the aforementioned leadership styles, personality trait, and thriving at work.

The leadership styles and personality traits of a leader enable the employees and the organization to move from one paradigm to another. The study has identified the effectiveness of both the leadership styles (authoritative and laissez-faire) and the personality trait (conscientiousness) in a service sector (education). These findings need to be subjected to future research to determine their further application in our educational environments. We hope that this work generates new research focusing on leadership styles and personality traits, leading to individuals thriving in the workplace in the service sector, especially in education.

## Figures and Tables

**Table 1:** Mean, standard deviations (SD), and correlations.

Variables	Mean	SD	1	2	3	4	5	6	7
1. Age	32.34	7.47							
2. Education	14.54	1.30	0.13 *						
3. Experience	4.30	4.11	−0.04	−0.12					
4. Authoritative	3.78	0.72	−0.09	0.02	0.04	**(0.83)**			
5. Laissez- Faire	3.23	0.79	−0.08	−0.01	−0.04	−0.21 **	**(0.79)**		
6. Conscientiousness	3.65	0.87	−0.01	−0.02	−0.07	0.07	0.02	**(0.94)**	
7. Thriving at Work	3.73	0.63	0.01	−0.04	−0.04	0.19 **	−0.14 *	0.13 *	**(0.83)**

Note: ** Correlation is significant at the 0.01 level (two-tailed). * Correlation is significant at the 0.05 level (two-tailed). Cronbach’s alphas are on the diagonal (bold highlighted).

**Table 2:** Regression analysis for the direct relationships of the study variables.

Variables	Model 1	Model 2	Model 3	Model 4	Model 5
Age	0.00	0.00	−0.00	−0.00	0.00
Marital status	0.09	0.06	0.00	0.09	0.06
Education	−0.00	−0.00	−0.01	−0.02	−0.01
Job experience	−0.00	−0.00	−0.00	−0.00	−0.00
Authoritative		0.14 **			0.12 *
Laissez-Faire			−0.13 *		−0.11 *
Conscientiousness				0.09 *	0.08 *
R square	0.01	0.03	0.05	0.11	0.50
Δ R square		0.02	0.02	0.06	0.39

* *p* < 0.05, ** *p* < 0.01.

**Table 3:** Moderation analayis using Process by Hayes for thriving.

Models	Β	se	t
Conscientiousness	0.84 **	0.16	5.13
Laissez-faire	0.72 **	0.18	3.93
Laissez-faire × Conscientiousness	−0.23 **	0.05	−4.70
Authoritative leadership × Conscientiousness	0.07	0.06	1.11

Note: ** *p* < 0.01.

## Data Availability

The datasets generated during and/or analyzed during the current study are available from the corresponding author on reasonable request.

## Abbreviations

MLQ	Multi-factor leadership questionnaire
SD	Standard Deviation
CFI	Comparative fit index
TLI	Tucker–Lewis index
IFI	Incremental fit index
RMSEA	Root mean square error of approximation
